# Towards Sulphite-Free Winemaking: A New Horizon of Vinification and Maturation

**DOI:** 10.3390/foods13071108

**Published:** 2024-04-04

**Authors:** Nicola Mercanti, Monica Macaluso, Ylenia Pieracci, Guido Flamini, Giulio Scappaticci, Andrea Marianelli, Angela Zinnai

**Affiliations:** 1 Department of Agriculture, Food and Environment, University of Pisa, Via del Borghetto 80, 56126 Pisa, Italy; nicola.mercanti@phd.unipi.it (N.M.); monica.macaluso@unipi.it (M.M.); giulio.scappaticci@phd.unipi.it (G.S.); andrea.marianelli@phd.unipi.it (A.M.); angela.zinnai@unipi.it (A.Z.); 2 Department of Pharmacy, Via Bonanno 6, 56126 Pisa, Italy; guido.flamini@unipi.it; 3 Interdepartmental Research Centre “Nutraceuticals and Food for Health”, University of Pisa, Via del Borghetto 80, 56126 Pisa, Italy

**Keywords:** sulphur dioxide, winemaking, oxygen, vinification, maturation

## Abstract

The complex dynamics between oxygen exposure, sulphur dioxide (SO_2_) utilization, and wine quality are of the utmost importance in wine sector, and this study aims to explore their fine balance in winemaking. As a common additive, SO_2_ works as an antiseptic and antioxidant. However, its excessive use has raised health concerns. Regulatory guidelines, including Council Regulation (EC) N° 1493/1999 and Commission Regulation (EC) No 1622/2000, dictate SO_2_ concentrations in wines. The increasing demand for natural preservatives is driving the search for alternatives, with natural plant extracts, rich in phenolic compounds, emerging as promising substitutes. In this context, Bioma Company has proposed alternative additives deriving from vineyard waste to replace SO_2_ during winemaking. Thus, the aim of the present work was to compare the compositional characteristics between the product obtained with the alternative vinification and the traditional one during the winemaking, as well as the aroma compositions of the final wines. After a year of experimentation, the wines produced with Bioma products showed compositional characteristics comparable to their traditional counterparts. Notably, these wines comply with current legislation, with significantly reduced total sulphur content, allowing their designation as “without added sulphites”. Bioma products emerge as potential catalysts for sustainable and health-conscious winemaking practices, reshaping the landscape of the industry.

## 1. Introduction

The winemaking process, spanning from grape harvesting to the culminations of the fermentation and maturation phases, exposes wine to oxygen at various stages [1], which, although plays a key role in facilitating microbial development and polymerization of phenolic components, may have both positive and negative effects [2,3,4]. Limited and controlled amounts of oxygen introduced through micro-oxygenation may enhance aromas and reduce green and vegetal notes in wines [4,5,6]. Conversely, excessive exposure to oxygen may lead to an undesirable loss of colour and aroma [2,7]. To counteract these negative effects, the use of sulphur dioxide (SO_2_) during wine production has became crucial to mitigate undesirable outcomes [8,9,10]. The use of SO_2_ as a food preservative is regulated by the European authority. Regarding wine, Council regulation (EC) N° 1493/1999 of 17 May 1999 and Commission Regulation (EC) No 1622/2000 of 24 July 2000 have established a limited total concentration of SO_2_ up to 160 mg/L in red wines, and 210 mg/L in white and rosé wines [11]. Sulphur dioxide is a longstanding additive in winemaking thanks to its antiseptic, antioxidant, and antimicrobial properties [12,13]. Indeed, microbiologically, SO_2_ exerts an important antiseptic function that has proven to be specifically selective towards both acetic and lactic acid bacteria, and it also helps to determine the yeast population present [14], holding significance for both producers and consumers [10]. An excessive consumption of sulphites, however, can elicit symptoms such as headaches, nausea, stomach irritation, and respiratory distress, especially in asthmatic subjects [11,15,16]. Moreover, an overabundance of SO_2_ during the winemaking process can induce changes in the sensory characteristics of the final product [10,17]. To address these concerns, permissible concentrations of this additive in wines have been progressively reduced. The wine industry is increasingly compelled to minimize or eliminate SO_2_, particularly in the production of organic wines. This aspect highlights the need for novel and healthier strategies, prioritizing both the consumer well-being and the quality of the final wine product [10,18]. Several techniques have been employed to regulate the use of SO_2_ in the winemaking process, such as the application of high-pressure pulses [19], ultraviolet irradiation [20], and electric fields [21], or the development of alternative additives like lysozyme [22], ascorbic acid [23], glutathione [24], and plant extracts [25], or colloidal silver nanoparticles [26]. Nowadays, there is a growing consumer demand for foods with natural preservatives, inspiring interest in alternatives to chemical additives [27,28]. In the field of wine production, natural plant extracts have emerged as a promising alternative to sulphides. These extracts, rich in phenolic compounds recognized for their antioxidant and antimicrobial properties [29], are being explored as potential substitutes for sulphides in wine production. Indeed, polyphenols, acting as scavengers of free radicals and natural metal chelators, are widely employed in the food industry to preserve oxidative stability and extend products’ shelf life [30]. The demonstrated effectiveness of phenolic compounds and their derivatives in preventing autoxidation adds appeal to these natural preservatives [31,32,33,34]. In this context, some authors have successfully produced wines with a sensory profile similar to those produced with SO_2_ by substituting this preservative with extracts derived from winery by-products, such as grape seeds and stems rich in antioxidant compounds [35]. Wines produced with natural preservatives are seen as more competitive on the global market, mainly thanks to their numerous positive aspects, such as the prevention of degenerative diseases and their antimicrobial activity [36]. Nevertheless, further studies are necessary to understand the effects of plant extracts on the quality and sensory properties of the final wine products [37,38,39]. 

Thus, the aim of the present work was to evaluate the technological possibility of producing a wine with natural additives obtained from vineyard waste material, without compromising the final product. This is where the BIOMA SA Company (Quartino, Switzerland) comes in, offering alternative additives that can replace sulphur dioxide during the winemaking process. These alternative additives represent wine sector by-products constituted by red grapes pomace, characterized by a relevant content of polyphenols. In the present work, the chemical parameters (alcohol content, pH, acidity, phenolic composition, sulphur dioxide, and volatile composition) were evaluated during the winemaking and ageing processes in wines produced by traditional and alternative vinification, aged in both wood and steel vessels. Moreover, the aroma compositions of the musts and final wines were also assessed.

## 2. Materials and Methods

### 2.1. Wine Samples and Winemaking

The analysed wine samples, obtained from Sangiovese grapes, were produced by the La Cura winery in Massa Marittima (Italy). In the present study, a comparison between traditional and alternative winemaking, called Bioma, was carried out. The products of both the vinification procedures were obtained starting from musts with the same characteristics, shown in Table 1.

Bioma winemaking process (described in Table 2) differed from the traditional one only for the addition of the natural additives Epyca Red1, Red2, Red3, and ML-A, and ML-B, replacing the sulphur dioxide. The natural additives were supplied by the BIOMA SA Company (Quartino, Switzerland), while yeast and lactic bacteria by Lallemand Inc. Italia (Castel D’Azzano (VR), Italy). Conversely, yeast strains and malolactic bacteria responsible for alcoholic and malolactic fermentations were the same for both the winemaking processes in order to reduce the variables that could affect the final beverage. A volume of 10 hectolitres (hL) of product was vinified in two steal tanks for the entire fermentation period (from 24 September to 4 December) and the alcoholic fermentation temperature was controlled at 20 °C. The wines produced with the described vinification procedures were finally subjected to the wine ageing process in wood and steel vessels, obtaining four different products: traditional wine aged in wood (TW), traditional wine aged in steel (TS), Bioma wine aged in wood (BW), and Bioma wine aged in steel (BS).

### 2.2. Chemical Analyses

All the chemical determinations necessary for the characterization of wines (sugar content (hexoses g/L), titratable acidity (tartaric acid g/L), pH, L-malic acid (g/L), alcohol content (% *v*/*v*), AVN (net volatile acidity (g/L acetic acid)), were carried out according to the OIV (International Organisation of Vine and Wine) methods [40], while total phenols (g/L catechins) were measured using the Folin–Ciocalteu colorimetric assay, modified as follows: 1 mL of sample (previously diluted 1:10 with deionized water), 5 mL of Folin−Ciocalteu reagent, 15 mL of 20% sodium carbonate, and 79 mL of deionized water were mixed in a 100 mL glass flask, and after 30 min of incubation at room temperature, the absorbance of the samples was measured at 765 nm against blank. Total phenols content was expressed as g/L of catechin. Total anthocyanins (g/L malvin) and bleachable anthocyanins (g/L malvin) were evaluated following the hydrochloric acid method and the bisulphite bleaching method reported by Aleixandre-Tudo et al., respectively [41].

The contents of free and total sulphur dioxide were evaluated using an iCubio iMagic M9 analyser (Shenzhen iCubio Biomedical Technology Co., Ltd., Shenzhen, China), operating in complete automation. This system automatically pipettes the reagents and samples into the cuvette, allowing their reaction at a controlled temperature. After the incubation the absorbance at a specific wavelength is reading, and then the analyte concentrations are calculated with a calibration method [42]. The reagents used were Enzytec™ Liquid SO_2_-free Cod. E8610 and Enzytec™ Liquid SO_2_-total Cod. E8600. All the reagents and standards were purchased from R-Biopharm AG (Darmstadt, Germany). 

### 2.3. Headspace Solid-Phase Microextraction and GC-MS Analyses

The volatile emissions of the wine samples were analysed in triplicate by using Headspace Solid-Phase Microextraction (HS-SPME). For the analysis, 25 mL of each sample was placed in a 50 mL glass flask, covered with aluminium foil, and left to equilibrate for almost 30 min at room temperature. Then, the headspace was sampled for 5 min using a Supelco SPME device equipped with a divinylbenzene/carboxen/polydimethylsiloxane (DVB/CAR/PDMS) fibre (100 µm, Supelco analytical, Bellefonte, PA, USA), which was preconditioned following the manufacturer’s instructions. Once the sampling time was finished, the fibre was injected into the gas chromatography-mass spectrometry analyses apparatus (Agilent Technologies Inc., Santa Clara, CA, USA) equipped with an Agilent HP-5MS capillary column (30 m × 0.25 mm; coating thickness 0.25 µm) and an Agilent 5977B single quadrupole mass detector. The GC-MS analyses and peak identification were accomplished according to Pieracci et al. [32].

### 2.4. Statistical Analyses

One-way analysis of variance was performed using CoStat, Version 6.451, CoHort 6.0 Software (Pacific Grove, CA, USA) to assess the presence of significant differences among the investigated samples on the compositional parameters, while with JMP Pro statistical package (SAS Institute; Cary, NC, USA) was used to evaluate the presence of significant differences in the relative content of the identified chemical classes of the volatile profiles. For both the analyses, the means were separated by Tukey’s post hoc test using a *p* ≤ 0.05. Each analysis was performed in triplicate.

The complete composition of the aroma profile of the analysed wine samples was also subjected to multivariate statistical analyses by using principal component analysis (PCA) and hierarchical cluster analysis (HCA) methods through employing JMP Pro statistical package (SAS Institute; Cary, NC, USA).

## 3. Results and Discussion

The present work aimed to verify the technological possibility of producing quality wines without the addition of sulphites. Nowadays, although there are several possibilities for the obtainment of wines without added sulphites, the obtained products do not show characteristics comparable to those of traditional ones [43,44]. The quality of wine is defined by several parameters, including both the compositional characteristics and the aroma profile. To discriminate a good quality wine, it is important that it features a low volatile acidity, a good structural component, as well as good chromatic characters [45]. For this reason, the products obtained from Bioma winemaking process were analysed for their compositional and aroma characters and compared with the traditional ones. 

### 3.1. Bioma Products Compositions

The compositional characteristics of the formulations of the Epyca line, developed by Bioma Company, were assessed and the data are reported in Table 3. From a chemical point of view, some components useful for defining the extract, e.g., total phenols, pH, dry extract, and titratable acidity, were evaluated. In addition, the possibility that the detected compounds could significantly alter the compositional characteristics of the product they were added to (grapes, must under fermentation, wine before or after the course of malolactic fermentation) was assessed. The Epyca formulations dosage of use did not significant alter the composition of both raw material (grape or must) and finished product, neither in terms of alcohol content nor of phenolic content, as the average contribution accounted for almost 1 mg of phenols per litre of wine. Since organic acid content is considered to be a concern, its values were also assessed, resulting in modest amounts, as also confirmed by the pH values, which were comprised between 4.5 and 7.

### 3.2. Total and Free Sulphur Dioxide

**Figure 1:**
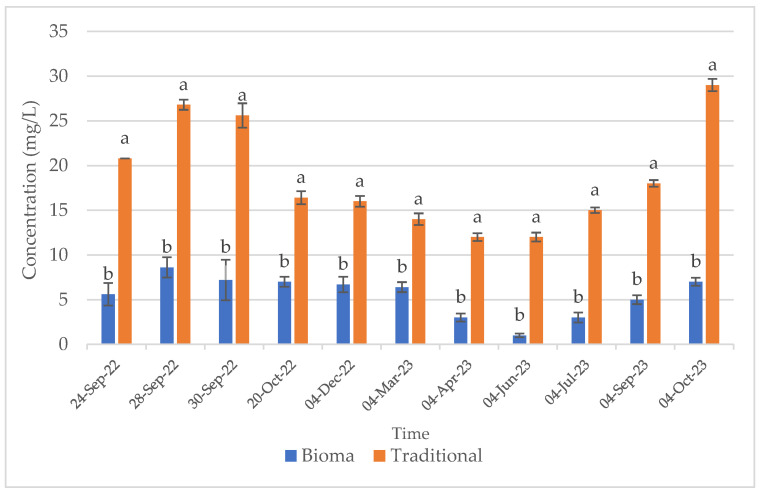
Free sulphur dioxide trend during winemaking (24 September 2022–4 December 2022) and ageing (4 December 2022–4 October 2023) of traditional and Bioma wines. Error bars represent standard deviation values (n = 3). Letters indicate statistically significant differences between Bioma and traditional samples at the same time.

**Figure 2:**
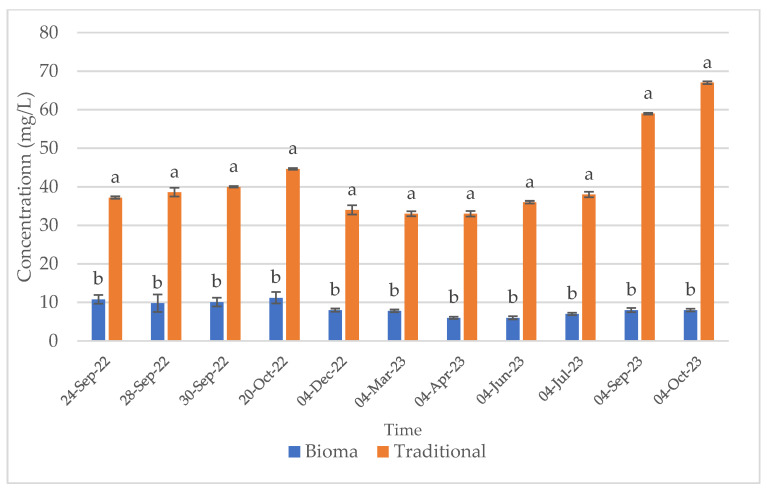
Total sulphur dioxide trend during winemaking (24 September 2022–4 December 2022) and ageing (4 December 2022–4 October 2023) of traditional and Bioma wines. Error bars represent standard deviation values (n = 3). Letters indicate statistically significant differences between Bioma and traditional samples at the same time.

Sulphur dioxide is the most used additive in winemaking, since, thanks to its antioxidant potential, it is able to protect the product from various oxidative reactions [46], besides to be a potent antimicrobial agent [13]. However, due to the numerous adverse effects of this additive, nowadays consumer preferences are increasingly directed toward the use of natural products, which are considered to be safer and healthier. The wines produced in this study with both of the vinification methods comply with the current legislation in terms of total sulphide dioxide. However, the substitution of this additive with the natural extracts obtained from vineyard waste material (Bioma Company) allowed us to obtain wines with significantly reduced total and free sulphur dioxide contents, enabling their designation as “*without added sulphites*”. The free and total sulphur dioxide trends during winemaking and maturation of the traditional and Bioma wines are reported in Figure 1 and Figure 2, respectively.

### 3.3. Alcoholic and Malolactic Fermentation Trends

During winemaking, the yeast strains present in the must, both those autochthonous naturally occurring on the grapevines and those selected, are responsible for alcoholic fermentation, during which sugars are converted into ethanol and carbon dioxide [45]. The alcoholic is a step of pivotal importance, and the monitoring of glucose and ethanol during the fermentation process is important to control the quality of the wine. Usually, after alcoholic fermentation, red wines undergo malolactic fermentation, which consists in the transformation of malic acid into lactic acid, as it is carried out by lactic bacteria of the *Lactobacillus*, *Leuconostoc*, and *Pediococcus* genera. This fermentation determines the elimination of the sour taste caused by malic acid and the increase in lactic acid, which are responsible for a smooth and round taste, in turn due to the reduced fixed acidity [47].

**Figure 3:**
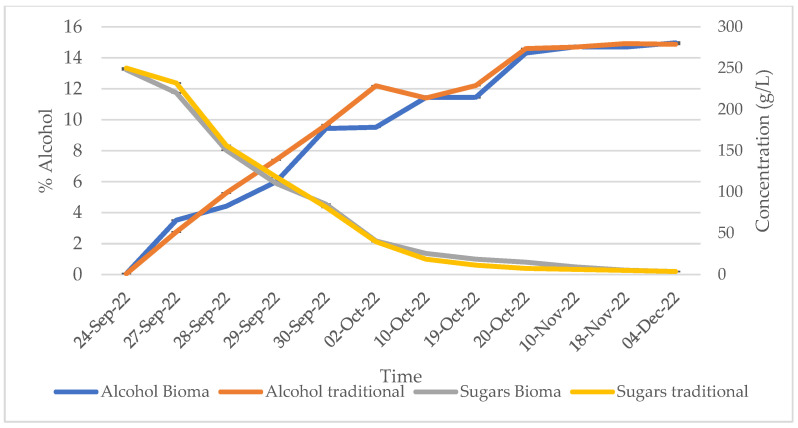
Trend of sugars and alcohol during alcoholic fermentation of traditional and Bioma wines.

**Figure 4:**
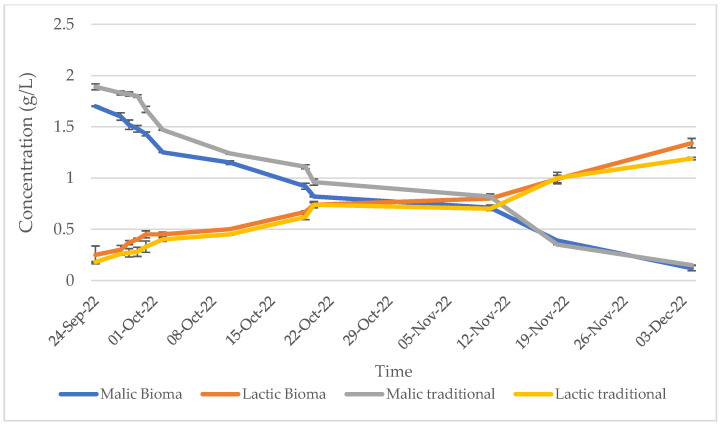
Trend of malic and lactic acids during the malolactic fermentation of traditional and Bioma wines.

In the present study, the trends of sugars and alcohols during alcoholic fermentation (Figure 3), as well as of malic and lactic acids during the malolactic fermentation (Figure 4), were assessed and were found to be similar for the wines produced with traditional and alternative winemaking processes, evidencing no significant alterations in the fermentative processes. In detail, at the end of the fermentation, sugars were detected at a concentration of almost 3 g/L and alcohol at 15% *v*/*v* in both the traditional and Bioma wines, while malic acid and acetic acid accounted for 0.12 and 0.15 g/L, and 1.34 and 1.19 in Bioma and traditional wines, respectively.

### 3.4. Volatile Acidity

**Figure 5:**
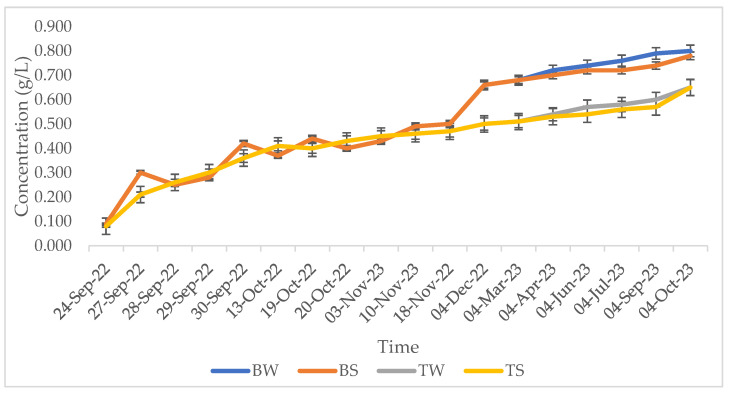
Volatile acidity of the traditional and Bioma wines during wine ageing in wood and steel.

Volatile acidity, expressed as acetic acid content (g/L), represents a parameter routinely used as an indicator of wine degradation [48]. As expected, the volatile acidity of both the traditional and alternative wines (Figure 5) showed a tendency to increase during the wine fermentation (from September to December) and ageing (from December to October) processes in both wood and steel vessels, without showing differences between the two types of refinements. Indeed, among the different acids contributing to volatile acidity, acetic acid is produced as a by-product of the alcoholic fermentation, and also as a product of sugar metabolization by acetic and lactic bacteria [49]. Moreover, volatile acidity increases with time due to the oxidation of ethanol and the extraction of phenolic compounds and volatile carboxylic acids from wood during the ageing process. Conversely, the type of vinification seemed to influence the volatile acidity, which was found to be 0.12 g/L higher in the wine produced with the Bioma additives; however, this wine showed a content lower than the maximum acceptable threshold of 1.2 g/L, as stated by OIV. The higher volatile acidity of the Bioma wines could be explained by a greater metabolic activity of bacteria, since sulphur dioxide, besides exerting antioxidant activity, also explicates antimicrobial activity, which could be the reason for the lower volatile acidity concentrations in the traditional wines.

### 3.5. Total Phenolics and Total and Bleachable Anthocyanins Content

Phenolic compounds are of the utmost importance in the quality of red wine due to their strong influence on colour, mouthfeel, and ageability. In the present study, the total phenolic contents, expressed as g/L of catechins, showed a typical trend during the winemaking process. Indeed, their content increased during the initial months, coinciding with the fermentation process, during which there was also an increase in both the alcohol content (Figure 1), characterized by a strong solubilising power on polar compounds [50], and the temperature, which also plays an important role in the extraction of phenolic compounds. In the following months of wine maturation, instead, these secondary metabolites underwent to a decrease that could be explained by their ability to react with oxygen molecules. Phenolic compounds exhibited the same development in both the investigated vinification processes, and few differences between traditional and Bioma wines were observed (Table 4). The obtained results evidence a good management of the operative variables, such as temperature and oxygen control, which are able to significantly influence the stability of wine phenols. The performed assay was not able to discriminate among the different classes of phenolic compounds, but this trend could very likely be attributable to anthocyanins, which are phenolic compounds that, due to the presence of conjugated bonds in their structure, are responsible for the red or purple colour of wine. Indeed, several studies have demonstrated that their extraction reaches a maximum in the early stages of the fermentation and that the concentration drops thereafter [51]. This hypothesis was confirmed by the results of total and bleachable anthocyanins performed on the analysed wines, whose concentrations trend during the wine fermentation and ageing was comparable to that of the total phenolic compounds. However, the decrease in the concentration of anthocyanins varied depending on the vessel in which the wine underwent the maturation process, being more marked in the wood barrels (Table 3). Indeed, barrels may be considered as an active vessels responsible for a remarkable oxygen transmission rate that plays a fundamental role in the ageing process, enhancing the oxidative reactions that influence the stability of anthocyanins [52]. These molecules are, in fact, very unstable in their free form and tend to interact with other phenolic compounds, including those from wood, and with low-molecular-weight compounds, which promote colour-stabilisation reactions; these reactions are favoured by the presence of oxygen, which plays an important role in the quality of the final wine.

### 3.6. Aroma Composition

Volatile compounds represent an important characteristic of wine products as they are responsible for the aroma, which is likely one of the most valuable quality feature able to influence consumer acceptability [53]. Wine aroma is conferred by the released volatile organic compounds (VOCs), the contents of which are influenced by the vineyard, fermentation, and ageing processes the wine undergoes [54], being responsible for primary, secondary, and tertiary aromas [53], respectively [5,55]. 

The complete chemical composition of the must and the wines refined in both steel and wood barrels obtained with traditional and alternative vinification methods are reported in Table 5. Overall, 34 compounds were identified, covering 99.4–100.0% of the whole volatile profiles. Undoubtedly, the class of non-terpene derivatives was the most representative. Within this class, esters, accounting for 55.9–82.1%, were detected as the most abundant compounds in all the analysed samples, even though significant differences in their contents were found. Indeed, as evidenced by the one-way ANOVA, their content was significantly higher in the must obtained by the both traditional and Bioma vinification processes. This result corroborate the literature, since ethyl esters of fatty acids, after being produced in amounts exceeding their equilibrium concentration [53] during the fermentation stage, undergo a reduction on behalf of long-chain alcohols and volatile fatty acids, which, instead, face an increase in their concentration [5]. 

Volatile esters are among the most relevant classes responsible for the fruity aromas of wine products, and they derive mainly from the metabolism of fatty acids through enzymatic pathways [5]. They are defined as compounds formed by the condensation of a hydroxyl group of a phenol or an alcohol and a carboxyl group from an organic acid, and they are produced by yeasts during alcoholic fermentation, as well as during the malolactic fermentation. These molecules, if present in proper concentrations, confer pleasant notes, contributing to fruity and floral attributes and improving the quality of wines with poor varietal aroma characters [55]. 

Among esters, ethyl octanoate (16.9–40.7%), which is responsible for pineapple and strawberry hints, was the most abundant both in musts and wines, and together with ethyl hexanoate and ethyl decanoate, undergoes a reduction during vinification. Conversely, ethyl acetate increases when passing from must to wine, reaching almost 10–12% of the wine profile. Acetate esters are known to provide pleasant fruity aroma attributes. However, high concentrations of some of them, such as ethyl acetate, can negatively influence the wine, imparting a varnish and/or nail polish aroma, and also affecting the perception of favourable fruity ethyl esters by showing a suppressive effect.

As previously reported, besides the reduction in esters during the vinification, an increase in long-chain alcohols and acids was also observed in the analysed samples. Higher alcohols represent the most important aroma contributors to wine as they are able to impart pleasant aromatic notes. Among the alcohols detected in the analysed wines, isoamyl alcohol, characterized by a marzipan scent [55]; phenylethyl alcohol, responsible for the rose aroma [55]; and amyl alcohol were the most representative compounds. Concerning acids, acetic acid was the only compound detected that belongs to this class, and although its content showed an increase passing from must to wine, it never exceeded 1.7%. This molecule, besides ethanol and glycerol, directly originates from glycolysis, which occurs during fermentation and is not responsible for the aroma bouquet of the beverage. However, it could affect the perception of flavours imparted by the volatile compounds [5].

These variations occur as a result of the ageing process, as well as due to the formation of the lees, which are also responsible for the removal of some unpleasant volatile phenols from the wine [5,55].

Beyond non-terpenes, monoterpenes in both their hydrocarbon and oxygenated forms were found in non-negligible amounts in Bioma wines. In particular, 4-terpineol represented the major component, accounting for 6.1 and 5.3% in the steel- and wood-refined wines, respectively.

The complete chemical compositions of the aroma profiles of the analysed samples were subjected to multivariate statistical analyses with principal component analysis (PCA) and hierarchical cluster analysis (HCA) methods. PCA was performed on a correlation data matrix of 6 × 34 (6 samples × 34 compounds = 204 data), selecting the two highest PCs obtained by the linear regressions. The total explained variance of 83.0% was covered by a PC1 of 62.0% and a PC2 of 21.0%. HCA was carried out by using Ward’s method on non-standardized data, with squared Euclidean distances employed as a measure of similarity. 

**Figure 6:**
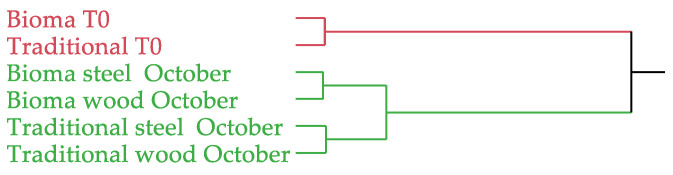
Dendrogram of the HCA performed on the volatile profile of the analysed samples.

The dendrogram of the HCA, as shown in Figure 6, evidenced a clear separation of the musts from the wines obtained with both the traditional and Bioma methods. Indeed, the former were plotted together to form the red cluster, while the wines constituted the green group. Within this latter group, a further separation based on the vinification process was evident. In fact, the Bioma samples, refined in both steel and wood barrels, were grouped in the first sub-group, while traditional wines in the second sub-group. 

**Figure 7:**
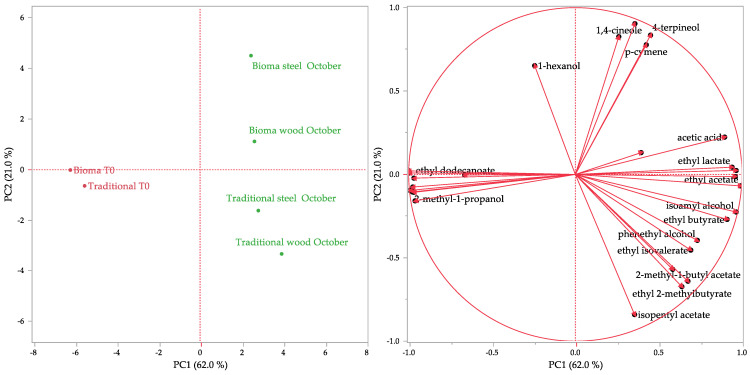
Score and loading plots of the PCA performed on the volatile profile of the analysed samples.

The same partitioning was also evidenced by the score plot of the PCA (Figure 7), in which the musts (Bioma T0 and traditional T0) were plotted in the bottom left quadrant, very close to the PC1-axys, while the wines were plotted in the right quadrants. Also observed in this case was a separation between the Bioma and traditional vinification outstood, since the wines produced with the alternative protocol were represented in the upper part while those obtained with the traditional method were represented in the lower one. This clear separation between them was determined by the greater content of 4-terpineol and *p*-cymene in the Bioma wines, the vectors of which were directed toward the uppermost area of the upper right quadrant of the loading plot. Conversely, the two types of aging of the wines produced with both the vinification methods seem to not be responsible for the differences in the aroma composition, as the Bioma samples were plotted in the upper right quadrants while traditional wines were plotted in the bottom right ones.

Although the statistical treatment evidenced a separation of the samples, it was possible to highlight that the vinification method did not dramatically affect the aroma composition of the products. Furthermore, the increased content of monoterpenes in the Bioma samples could be considered positive, as they contribute to the aroma of the wine with pleasant notes such as floral and citrus [56].

## 4. Conclusions

In conclusion, it is clear that the use of Bioma products provides a practicable method to produce wines without added sulphites while maintaining essential compositional attributes similar to traditional winemaking practices. This approach represents a significant advance in meeting consumer demands for healthier and safer wine options. Furthermore, the resulting wines exhibit a similar aromatic profile, with the Bioma wines in particular showing a marked increase in monoterpenes, thereby exhibiting enhanced aromatic complexity.

It is remarkable that the oxidation process, monitored through volatile acidity, is comparable for both vinification processes. However, despite the promising results of the Bioma approach, it is essential to emphasise the indispensable role of sulphur dioxide in winemaking and the absence of equally effective alternatives. While showing some potential, the Bioma method has not yet achieved the effectiveness of traditional sulphite-based techniques. Therefore, while the exploration of alternative additives is commendable, it underlines the continued need for research and innovation in the pursuit of more sustainable and healthier winemaking practices.

These results underline the importance of exploring new strategies that prioritise consumer welfare while safeguarding wine quality. The Bioma approach serves as a beacon in this endeavour, signalling a potential shift toward a more sustainable and health-conscious wine industry.

## Figures and Tables

**Table 1:** Must composition at starting time.

Must Composition	Concentration
Alcohol (% *v*/*v*)	0.06 ± 0.02
Sugar (g/L)	248 ± 12
pH	3.5 ± 0.01
Titratable acidity (g/L)	2.39 ± 0.11
Volatile acidity (g/L)	0.09 ± 0.03
Malic acid (g/L)	1.7 ± 0.13
Lactic acid (g/L)	0.25 ± 0.05

**Table 2:** Complete procedures for traditional and Bioma winemaking.

Winemaking Steps	Traditional Winemaking	Bioma Winemaking
Harvest	Addition of 5 g/hL K_2_S_2_O_5_	Addition of Epyca Red1 (1 L/3000 kg)
Alcoholic fermentation (a.f.)	Persy yeasts (5 g/hL)	Persy yeasts (5 g/hL)
Pumping over	Two a day	Two a day
Beginning of malolactic fermentation (m.l.f)	Malolactic bacteria prime 48 h after the beginning of A.F.	Malolactic bacteria prime 48 h after the beginning of A.F.
Racking	Addition of 8 g/hL K_2_S_2_O_5_	Addition of Epyca Red 2 (1 L/3000 kg) and ML-A and ML-B
Wine maturation	Wood and steel	Wood and steel
Filtration	-	-
Bottling	-	Addition of Epyca Red 3 (1 L/30 Q)

**Table 3:** Compositional characteristics of the formulations of the Epyca line developed by the Bioma company. Data are expressed as means ± confidence value (n = 3).

Sample	Dry Extract (g/L)	Total Phenols (Catechins g/L)	Alcohol (vol. %)	Total Phenols (Gallic A. g/L)	SO_2_ Tot. (mg/L)	pH	Titratable Acidity (g/L)
Red1	0.6 ± 0.1	0.37 ± 0.05	7.75 ± 0.20	0.12 ± 0.04	12.8 ± 0.9	5.0 ± 0.1	0.07 ± 0.01
Red 2	1.9 ± 0.1	0.64 ± 0.05	8.63 ± 0.1	0.27 ± 0.09	12.8 ± 1.8	4.8 ± 0.2	0.09 ± 0.01
Red 3	1.3 ± 0.1	0.66 ± 0.01	7.97 ± 0.05	0.25 ± 0.01	19.2 ± 2.1	4.5 ± 0.2	0.09 ± 0.01
ML-A	0.97 ± 0.10	0.69 ± 0.10	38.83 ± 0.90	0.27 ± 0.01	16.2 ± 1.1	4.8 ± 0.2	0.05 ± 0.01
ML-B	16.56 ± 0.40	2.09 ± 0.10	7.70 ± 0.10	0.14 ± 0.01	8.2 ± 0.9	7.2 ± 0.2	0.01 ± 0.00

**Table 4:** Content of total phenols and total and bleachable anthocyanins detected in all the analysed samples.

Sampling Time	Total Phenols (g/L Catechin)				Total Anthocyanins (mg/L Malvin)				Bleachable Anthocyanins (mg/L Malvin)			
	Bioma		Traditional		Bioma		Traditional		Bioma		Traditional	
24-Sept	1.17 ± 0.03 ^B^		1.72 ± 0.02 ^A^		139.65 ± 1.88 ^A^		134.75 ± 19.79 ^A^		52.94 ± 0.62 ^B^		70.49 ± 0.94 ^A^	
27-Sept	2.88 ± 0.01 ^B^		3.11 ± 0.03 ^A^		193.85 ± 0.47 ^B^		230.42 ± 1.41 ^A^		61.25 ± 4.95 ^B^		172.38 ± 9.89 ^A^	
28-Sept	3.16 ± 0.02 ^B^		3.28 ± 0.01 ^A^		194.51 ± 1.41 ^B^		273.32 ± 0.94 ^A^		141.31 ± 0.62 ^B^		209.13 ± 14.84 ^A^	
29-Sept	3.25 ± 0.01 ^B^		3.4 ± 0.02 ^A^		205.49 ± 4.70 ^B^		287.28 ± 2.82 ^A^		144.38 ± 6.18 ^B^		266.88 ± 16.08 ^A^	
30-Sept	3.44 ± 0.01 ^B^		3.55 ± 0.03 ^A^		291.27 ± 2.82 ^B^		367.08 ± 1.88 ^A^		209.13 ± 16.08 ^A B^		275.19 ± 19.18 ^A^	
13-Oct	3.61 ± 0.02 ^A^		3.17 ± 0.01 ^B^		294.6 ± 1.88 ^B^		402.99 ± 0.94 ^A^		203.88 ± 9.89 ^B^		295.75 ± 13.61 ^A^	
19-Oct	3.9 ± 0.02 ^A^		3.42 ± 0.04 ^B^		423.94 ± 0.47 ^B^		445.22 ± 0.47 ^A^		289.63 ± 1.23 ^A^		292.69 ± 8.04 ^A^	
20-Oct	3.97 ± 0.02 ^A^		3.58 ± 0.02 ^B^		364.42 ± 2.82 ^A^		360.7 ± 10.72 ^A^		304.5 ± 1.23 ^A^		255.06 ± 10.51 ^B^	
03-Nov	4.6 ± 0.14 ^A^		4.09 ± 0.02 ^B^		369.74 ± 1.88 ^B^		356.44 ± 1.88 ^A^		302.75 ± 3.71 ^A^		273 ± 7.42 ^B^	
10-Nov	4.83 ± 0.11 ^A^		4.4 ± 0.05 ^B^		313.69 ± 4.33 ^B^		328.94 ± 2.21 ^A^		313.98 ± 1.92 ^A^		262.94 ± 19.18 ^B^	
18-Nov	3.8 ± 0.01 ^A^		3.78 ± 0.01 ^A^		287.58 ± 27.79 ^A^		286.58 ± 0.33 ^A^		300 ± 2.22 ^A^		270.55 ± 7.42 ^A B^	
04-Dec	3.64 ± 0.01 ^A^		3.64 ± 0.01 ^A^		299.95 ± 4.94 ^A^		293 ± 23.04 ^A^		280 ± 2.78 ^A^		204.53 ± 0.68 ^B^	
	BW	BS	TW	TS	BW	BS	TW	TS	BW	BS	TW	TS
04-March	3.02 ± 0.11 ^A^	3.02 ± 0.11 ^A^	2.73 ± 0 ^A^	2.73 ± 0 ^A^	297.85 ± 13.54 ^A^	297.85 ± 3.53 ^A^	273.45 ± 0.18 ^A^	273.45 ± 0.18 ^A^	250 ± 1.98 ^A^	250 ± 1.98 ^A^	199.24 ± 0.37 ^A^	199.24 ± 0.37 ^A^
04-Apr	2.73 ± 0.01 ^B^	2.89 ± 0.17 ^A^	2.62 ± 0.06 ^A B^	2.7 ± 0.13 ^A^	288 ± 5.6 ^A^	288 ± 5.6 ^A^	265.23 ± 0.32 ^A^	265.23 ± 0.32 ^A^	243 ± 0.00 ^A^	243 ± 0.01 ^A^	190 ± 0.00 ^A^	190 ± 0.00 ^A^
04-Jun	2.46 ± 0.01 ^B^	2.56 ± 0.2 ^A^	2.36 ± 0.01 ^B^	2.54 ± 0.09 ^A^	281 ± 10.1 ^A^	285 ± 10 ^A^	255.13 ± 4.32 ^B^	264.2 ± 4.23 ^A^	210 ± 4.10 ^A^	210 ± 4.10 ^A^	183 ± 0.00 ^A^	183 ± 0.00 ^A^
04-July	2.2 ± 0.05 ^A^	2.22 ± 0.1 ^A^	2.11 ± 0.05 ^A^	2.12 ± 0.05 ^A^	235.94 ± 0.66 ^A^	256 ± 5.3 ^A^	199.5 ± 1.88 ^B^	263.7 ± 5.16 ^A^	187 ± 0.01 ^B^	205.6 ± 6.7 ^A^	167 ± 0.92 ^A B^	174.6 ± 3.54 ^A^
04-Sept	2.18 ± 0.03 ^A^	2.2 ± 0.07 ^A^	2.2 ± 0.01 ^A^	2.09 ± 0.1 ^B^	209.14 ± 0.47 ^B^	248 ± 4.3 ^A^	188 ± 0.47 ^B^	263 ± 6.23 ^A^	180.91 ± 3.2 ^B^	200.8 ± 4.54 ^A^	158 ± 0.33 ^A^	150.4 ± 0.98 ^A^
04-Oct	2.15 ± 0.05 ^A^	2.15 ± 0.06 ^A^	2.13 ± 0.08 ^A^	2 ± 0.06 ^B^	182.3 ± 0.65 ^B^	224 ± 7.9 ^A^	187.5 ± 0.80 ^B^	240.5 ± 0.98 ^A^	180 ± 3.898 ^A B^	190.7 ± 10.2 ^A^	150 ± 5.25 ^A^	145.4 ± 1.45 ^A B^

Superscript uppercase letters (A–B) indicate statistically significant differences in the relative abundances of the chemical compounds among the samples, *p* < 0.05.

**Table 5:** Complete chemical composition of the musts and the wines refined in both steel and wood barrels obtained with traditional and alternative vinification methods.

Compounds	l.r.i. ^1^	Class	Relative Abundance ± Standard Deviation (n = 3)					
			Bioma T0	Bioma Steel October (BS)	Bioma Wood October (BW)	Traditional T0	Traditional Steel October (TS)	Traditional Wood October (TW)
acetic acid	610	nt	0.5 ± 0.22 ^C^	1.3 ± 0.09 ^AB^	1.7 ± 0.31^A^	0.2 ± 0.02 ^C^	1.2 ± 0.08 ^B^	1.2 ± 0.04 ^B^
ethyl acetate	612	nt	2.0 ± 0.05 ^D^	9.8 ± 0.04 ^C^	11.8 ± 0.79 ^AB^	1.8 ± 0.11 ^D^	11.3 ± 0.09 ^B^	12.7 ± 0.05 ^A^
2-methyl-1-propanol	625	nt	0.4 ± 0.06 ^B^	- ^2,C^	- ^C^	0.5 ± 0.04 ^A^	- ^C^	- ^C^
acetale	726	nt	1.5 ± 0.10 ^A^	- ^B^	- ^B^	- ^B^	- ^B^	- ^B^
isoamyl alcohol	736	nt	8.8 ± 0.48 ^C^	17.7 ± 0.54 ^B^	17.9 ± 1.83 ^B^	10.6 ± 0.69 ^C^	21.5 ± 0.53 ^A^	22.4 ± 0.66 ^A^
amyl alcohol	739	nt	3.0 ± 0.03 ^B^	6.4 ± 0.61 ^A^	8.4 ± 1.62 ^A^	2.9 ± 0.34 ^B^	7.2 ± 0.42 ^A^	7.5 ± 0.34 ^A^
2,3-butanediol	790	nt	0.7 ± 0.11	0.7 ± 0.16	0.8 ± 0.28	0.3 ± 0.07	3.3 ± 2.55	2.2 ± 1.16
ethyl butyrate	803	nt	0.2 ± 0.01 ^D^	0.6 ± 0.06 ^AB^	0.5 ± 0.16 ^BC^	0.3 ± 0.04 ^CD^	0.8 ± 0.06 ^A^	0.8 ± 0.06 ^A^
ethyl lactate	813	nt	- ^D^	1.5 ± 0.01 ^B^	2.0 ± 0.19 ^A^	- ^D^	1.1 ± 0.07 ^C^	2.0 ± 0.30 ^A^
ethyl 2-methylbutyrate	849	nt	- ^C^	- ^C^	0.1 ± 0.01 ^B^	- ^C^	0.1 ± 0.01 ^B^	0.3 ± 0.02 ^A^
ethyl isovalerate	852	nt	- ^D^	0.2 ± 0.01 ^C^	- ^D^	- ^D^	0.3 ± 0.02 ^B^	0.5 ± 0.01 ^A^
1-hexanol	903	nt	0.4 ± 0.05 ^A^	0.5 ± 0.07 ^A^	0.4 ± 0.18 ^A^	0.4 ± 0.01 ^A^	0.5 ± 0.01 ^A^	- ^B^
isopentyl acetate	877	nt	5.4 ± 0.15 ^E^	5.4 ± 0.01 ^E^	6.0 ± 0.04 ^D^	6.9 ± 0.16 ^C^	7.4 ± 0.09 ^B^	7.9 ± 0.12 ^A^
2-methyl-1-butyl acetate	880	nt	1.0 ± 0.03 ^D^	1.1 ± 0.05 ^CD^	1.3 ± 0.15 ^ABC^	1.2 ± 0.1 ^BC^	1.3 ± 0.01 ^AB^	1.5 ± 0.01^A^
ethyl hexanoate	1000	nt	14.4 ± 0.16 ^B^	9.4 ± 0.27 ^C^	9.3 ± 0.56 ^C^	16.1 ± 0.23 ^A^	9.9 ± 0.14 ^C^	9.7 ± 0.76 ^C^
hexyl acetate	1012	nt	0.3 ± 0.01^B^	- ^C^	- ^C^	0.4 ± 0.00 ^A^	- ^C^	- ^C^
1,4-cineole	1015	om	- ^B^	0.2 ± 0.01 ^A^	- ^B^	- ^B^	- ^B^	- ^B^
α-terpinene	1017	mh	- ^B^	1.0 ± 0.07 ^A^	- ^B^	- ^B^	- ^B^	- ^B^
*p*-cymene	1024	mh	- ^B^	2.0 ± 0.06 ^A^	2.3 ± 0.29 ^A^	- ^B^	- ^B^	- ^B^
γ-terpinene	1028	mh	- ^C^	0.9 ± 0.09 ^A^	0.3 ± 0.06 ^B^	- ^C^	- ^C^	- ^C^
terpinolene	1088	mh	- ^B^	1.0 ± 0.01 ^A^	- ^B^	- ^B^	- ^B^	- ^B^
ethyl heptanoate	1097	nt	0.2 ± 0.00	-	-	0.2 ± 0.00	-	-
nonanal	1105	nt	-	0.9 ± 0.73	-	0.1 ± 0.1	1.7 ± 1.66	-
phenethyl alcohol	1116	nt	4.9 ± 2.04 ^AB^	5.5 ± 0.28 ^AB^	4.8 ± 0.32 ^AB^	3.0 ± 0.08 ^B^	6.5 ± 0.86 ^AB^	7.9 ± 2.29 ^A^
methyl octanoate	1126	nt	0.1 ± 0.01 ^A^	- ^B^	- ^B^	0.1 ± 0.00 ^A^	- ^B^	- ^B^
4-terpineol	1177	om	- ^C^	6.1 ± 0.05 ^A^	5.3 ± 0.23 ^B^	- ^C^	0.2 ± 0.03 ^C^	0.2 ± 0.01 ^C^
diethyl succinate	1180	nt	- ^D^	1.3 ± 0.04 ^BC^	1.5 ± 0.01 ^AB^	- ^D^	1.0 ± 0.2 ^C^	1.7 ± 0.29 ^A^
ethyl octanoate	1198	nt	40.7 ± 0.82 ^A^	20.8 ± 0.65 ^B^	20.7 ± 1.12 ^B^	39.8 ± 0.98 ^A^	19.1 ± 1.07 ^BC^	16.9 ± 1.44 ^C^
isopentyl hexanoate	1252	nt	0.3 ± 0.04 ^B^	- ^C^	- ^C^	0.3 ± 0.02 ^A^	- ^C^	- ^C^
β-phenylethyl acetate	1258	nt	0.3 ± 0.02 ^A^	- ^C^	- ^C^	0.2 ± 0.01 ^B^	- ^C^	- ^C^
ethyl decanoate	1396	nt	13.2 ± 0.39 ^A^	5.5 ± 0.67 ^B^	5.0 ± 0.30 ^B^	13.4 ± 0.64 ^A^	4.9 ± 0.01 ^BC^	3.9 ± 0.05 ^C^
isoamyl octanoate	1446	nt	0.4 ± 0.02 ^A^	- ^B^	- ^B^	0.4 ± 0.04 ^A^	- ^B^	- ^B^
2-methylbutyl octanoate	1449	nt	0.1 ± 0.00	-	-	-	-	-
ethyl dodecanoate	1595	nt	1.1 ± 0.04 ^A^	0.3 ± 0.01 ^B^	- ^C^	1.0 ± 0.15 ^A^	0.1 ± 0.03 ^BC^	0.2 ± 0.09 ^BC^
**Chemical Classes**			**Bioma T0**	**Bioma Steel October**	**Bioma Wood October**	**Traditional T0**	**Traditional Steel October**	**Traditional Wood October**
Monoterpene hydrocarbons (mh)			- ^C^	4.9 ± 0.22 ^A^	2.6 ± 0.35 ^B^	- ^C^	- ^C^	- ^C^
Oxygenated monoterpenes (om)			- ^C^	6.3 ± 0.05 ^A^	5.3 ± 0.23 ^B^	- ^C^	0.2 ± 0.03 ^C^	0.2 ± 0.01 ^C^
Other non-terpene derivatives (nt)			99.9 ± 0.10 ^A^	88.9 ± 0.17 ^C^	92.2 ± 0.57 ^B^	100.1 ± 0.01 ^A^	99.2 ± 0.51 ^A^	99.3 ± 0.38 ^A^
*Alcohols*			*18.2 ± 1.32* ^C^	*30.8 ± 0.44* ^B^	*32.3 ± 0.99* ^B^	*17.7 ± 1.07* ^C^	*39.0 ± 2.46* ^A^	*40.0 ± 2.12* ^A^
*Acids*			*0.5 ± 0.22* ^C^	*1.33 ± 0.09* ^AB^	*1.7 ± 0.31* ^A^	*0.2 ± 0.02* ^C^	*0.12 ± 0.08* ^B^	*1.2 ± 0.04* ^B^
*Esters*			*79.7 ± 1.63* ^A^	*55.9 ± 1.43* ^B^	*58.2 ± 0.73* ^B^	*82.1 ± 1.17* ^A^	*57.3 ± 1.39* ^B^	*58.1 ± 1.70* ^B^
*Aldehydes*			*1.5 ± 0.10*	*0.9 ± 0.73*	*-*	*0.1 ± 0.01*	*1.7 ± 1.66*	*-*
Total identified (%)			99.9 ± 0.10	100.1 ± 0.10	100.1 ± 0.10	100.1 ± 0.01	99.4 ± 0.54	99.5 ± 0.37

^1^ Linear retention index experimentally determined on an HP-5MS capillary column. ^2^ Not detected. Superscript uppercase letters (A–E) indicate statistically significant differences in the relative abundances of the chemical classes among the samples, *p* < 0.05. Classes evidenced in italics belong to the class of the other non-terpene derivatives.

## Data Availability

The original contributions presented in the study are included in the article, further inquiries can be directed to the corresponding author.
